# Utility of pre-treatment FDG PET/CT–derived machine learning models for outcome prediction in classical Hodgkin lymphoma

**DOI:** 10.1007/s00330-022-09039-0

**Published:** 2022-08-25

**Authors:** Russell Frood, Matt Clark, Cathy Burton, Charalampos Tsoumpas, Alejandro F. Frangi, Fergus Gleeson, Chirag Patel, Andrew Scarsbrook

**Affiliations:** 1 Department of Nuclear Medicine, Leeds Teaching Hospitals NHS Trust, Leeds, UK; 2 Department of Radiology, Leeds Teaching Hospitals NHS Trust, Leeds, UK; 3 Leeds Institute of Health Research, University of Leeds, Leeds, UK; 4 Department of Haematology, Leeds Teaching Hospitals NHS Trust, Leeds, UK; 5 Department of Nuclear Medicine and Molecular Imaging, University Medical Center of Groningen, University of Groningen, Groningen, Netherlands; 6 Leeds Institute of Cardiovascular and Metabolic Medicine, University of Leeds, Leeds, UK; 7 Centre for Computational Imaging and Simulation Technologies in Biomedicine (CISTIB), School of Computing and School of Medicine, University of Leeds, Leeds, UK; 8 Medical Imaging Research Center (MIRC), University Hospital Gasthuisberg, KU Leuven, Leuven, Belgium; 9 Department of Radiology, Oxford University Hospitals NHS Foundation Trust, Oxford, UK

**Keywords:** Hodgkin disease: positron emission tomography computed tomography, Machine learning, progression-free survival

## Abstract

**Objectives:**

Relapse occurs in ~20% of patients with classical Hodgkin lymphoma (cHL) despite treatment adaption based on 2-deoxy-2-[^18^F]fluoro-d-glucose positron emission tomography/computed tomography response. The objective was to evaluate pre-treatment FDG PET/CT–derived machine learning (ML) models for predicting outcome in patients with cHL.

**Methods:**

All cHL patients undergoing pre-treatment PET/CT at our institution between 2008 and 2018 were retrospectively identified. A 1.5 × mean liver standardised uptake value (SUV) and a fixed 4.0 SUV threshold were used to segment PET/CT data. Feature extraction was performed using PyRadiomics with ComBat harmonisation. Training (80%) and test (20%) cohorts stratified around 2-year event-free survival (EFS), age, sex, ethnicity and disease stage were defined. Seven ML models were trained and hyperparameters tuned using stratified 5-fold cross-validation. Area under the curve (AUC) from receiver operator characteristic analysis was used to assess performance.

**Results:**

A total of 289 patients (153 males), median age 36 (range 16–88 years), were included. There was no significant difference between training (*n* = 231) and test cohorts (*n* = 58) (*p* value > 0.05). A ridge regression model using a 1.5 × mean liver SUV segmentation had the highest performance, with mean training, validation and test AUCs of 0.82 ± 0.002, 0.79 ± 0.01 and 0.81 ± 0.12. However, there was no significant difference between a logistic model derived from metabolic tumour volume and clinical features or the highest performing radiomic model.

**Conclusions:**

Outcome prediction using pre-treatment FDG PET/CT–derived ML models is feasible in cHL patients. Further work is needed to determine optimum predictive thresholds for clinical use.

**Key points:**

*• A fixed threshold segmentation method led to more robust radiomic features.*

*• A radiomic-based model for predicting 2-year event-free survival in classical Hodgkin lymphoma patients is feasible.*

*• A predictive model based on ridge regression was the best performing model on our dataset.*

**Supplementary Information:**

The online version contains supplementary material available at 10.1007/s00330-022-09039-0.

## Introduction

Hodgkin’s lymphoma (HL) is a haematopoietic malignancy characterised by the presence of Reed-Sternberg cells [1]. There are five different sub-classes of HL: nodular lymphocyte-predominant HL (NLPHL), and four under the umbrella category of classical HL (cHL): nodular sclerosing, mixed cellularity, lymphocyte-rich and lymphocyte-depleted. Ninety percent of HL cases are cHL [2]. NLPHL is often treated differently to cHL and is associated with more indolent progression [2]. Given the higher proportion of cHL cases, difference in treatment regimens and higher relapse rate in cHL compared to NLPHL, this paper will focus on cHL only [3].

Chemotherapy is the mainstay of frontline treatment of cHL; the most common regimes being doxorubicin (Adriamycin), bleomycin, vinblastine and dacarbazine (ABVD), or bleomycin, etoposide, doxorubicin (Adriamycin), cyclophosphamide, vincristine (Oncovin), procarbazine, and prednisone (BEACOPP) [4]. The treatment regime and number of cycles can vary depending on patient risk factors, disease stage and initial treatment response. Radiotherapy is used in patients with stage 1 or localised stage 2 disease or in residual bulky disease [4]. The gold standard imaging modality for staging and response assessment in HL is 2-deoxy-2-[^18^F]fluoro-d-glucose (FDG) positron emission tomography/computed tomography (PET/CT) [5]. Patients typically undergo PET/CT pre-treatment, following two cycles of chemotherapy (interim) and post-treatment. Interim PET/CT is used to guide treatment adaption, balancing the risk of chemotherapy-associated toxicity with maximising chances of event-free survival (EFS) [6]. Five-year survival in HL is approximately 86% [7]. However, even following complete metabolic response (CMR), approximately 20% of cHL patients will relapse with 72% of relapses occurring within the first 2 years of diagnosis [8]. The ability to identify patients at greater risk of relapse pre-treatment would allow upfront treatment stratification and could improve outcomes.

Previous studies assessing imaging parameters derived from baseline PET/CT for outcome prediction have mainly focused on metabolic tumour volume (MTV), total lesion glycolysis (TLG) and maximum or mean standardised uptake value (SUVmax and SUVmean) [9]. SUV is defined as the ratio of injected radioactivity within an image at a given timepoint when compared to the whole-body [10]. MTV is the volume of metabolically active segmented disease, with different segmentation techniques described [11]. The TLG is MTV multiplied by the SUVmean. Radiomics transforms images into mineable high-dimensional data permitting invisible feature extraction, analysis and modelling [12]. A limited number of studies using small sample sizes have demonstrated the potential of radiomic features in predicting progression-free survival (PFS) or overall survival (OS) in HL patients [13–16]. The aim of this work was to evaluate the performance of models using radiomic features derived from pre-treatment FDG PET/CT to predict 2-year EFS in cHL patients using a larger tertiary centre cohort of patients.

## Methods

This study adhered to the transparent reporting of a multivariable prediction model for individual prognosis or diagnosis (TRIPOD) guidelines (Supplemental Material 1).

### Patient selection

Retrospective review of radiology and clinical databases was performed to identify patients who had undergone FDG PET/CT for baseline staging of cHL at our institution between January 2008 and January 2018. This was chosen as the cut-off to allow a minimum of 2-year follow-up without confounding factors introduced by the COVID-19 pandemic. Patients were excluded if they were under 16 years of age, did not have cHL, had treatment prior to their staging PET/CT study, did not have measurable disease on PET/CT, had a concurrent malignancy or if the images were degraded or incomplete. Patients who had hepatic disease or had no measurable disease above 4.0 SUV were removed as this would influence the segmentation techniques used.

Patient age, ethnicity, disease stage, date of PET/CT, scanner model and protocol used, type and length of treatment, date of recurrence (confirmed by imaging or clinical examination), last clinical contact and length of follow-up or date of death were all recorded from electronic notes, from radiological records and from a regional haematological malignancy database. An event was defined as relapse, recurrence or death within the 2-year follow-up period. Due to missing clinical data, it was not possible to evaluate scoring systems such as the international prognostic score.

Informed written consent was obtained prospectively from all patients at the time of imaging for use of anonymised images in research and service development projects. As this was a retrospective study, not involving patient contact or the alteration of treatment, following discussion with the Research and Innovation Department at LTHT, it was agreed that this represented a service improvement project and was approved by the University of Leeds School of Medicine Research Ethics Committee (SoMREC).

### PET/CT acquisition

PET/CT studies were performed as part of routine clinical care using a standardised protocol. All patients fasted for 6h prior to administration of intravenous FDG (4MBq/kg). If serum blood glucose was > 10 mmol/L, the study was rescheduled following a clinical review of the patient’s diabetic control. Patients were scanned 1 h following FDG administration. Scans were acquired using a 16-slice Discovery STE PET/CT scanner (GE Healthcare) prior to June 2010; a 64-slice Philips Gemini TF64 scanner (Philips Healthcare) between June 2010 and October 2015; and 64-slice Discovery 690 or 710 scanners (GE Healthcare) after October 2015 (Table 1). Attenuation correction was performed using a CT component acquired with the following settings: 140 kV; 80 mAs; pitch 6; 3.75-mm slice thickness.

**Table 1:** Reconstruction parameters for the scanners used

Scanner	Matrix	Voxel size (column, row, slice thickness) (mm)	Reconstruction	Scatter correction	Random correction
GE Healthcare STE	128	4.6875 × 4.6875 × 3.27	OSEM	Convolution subtraction	Singles
GE Healthcare Discovery 690	192	3.65 × 3.65 × 3.27	VPFX	Model based	Singles
GE Healthcare Discovery 710	192	3.65 × 3.65 × 3.27	VPFX	Model based	Singles
Philips Gemini TF64	144 or 169	4 × 4 × 4	BLOB-OS-TF	SS-Simul	DLYD

*DLYD*, delayed event subtraction; *OSEM*, ordered subsets expectation maximisation; *SS-Simul*, single-scatter simulation; *VPFX*, Vue Point FX (3D Time of Flight); *BLOB-OS-TF*, a 3D ordered subset iterative TOF reconstruction algorithm (spherically symmetric basis function ordered subset)

### Image segmentation, feature extraction and machine learning analysis

A detailed methodology including detail of who performed the segmentation and interpretation of images is available in Supplemental Material 2. Two semi-automated segmentation techniques were used to contour the total lymphomatous disease within each study: the first using a fixed threshold of 4.0 SUV, and the second using a threshold of 1.5 × liver SUVmean. This method has been used in different cancer types (RTx v1.8.2, Mirada Medical) [17, 18]. Ten percent of cases were re-segmented using the same methodology following a 3-month washout period using Slicer (v4.11). These re-segmentations were used to investigate the robustness of the extracted radiomic features using different bin widths/bin numbers. Both the CT and PET images were resampled to a uniform voxel of 2 mm^3^. Features were extracted using PyRadiomics (v2.2.0) with 3935 features (PET/CT component × (shape features + first and second order features × number of filters)) extracted per segmentation technique for each patient (Supplemental Material 2: Table 1). Harmonisation to account for the different scanners was applied using the ComBat method (https://github.com/Jfortin1/ComBatHarmonization) [19].

The data was split into training and test cohorts stratified around 2-year EFS (2-EFS), age, sex, ethnicity, disease stage, having radiotherapy, having ABVD-based chemotherapy and being treated as advanced disease using scikit-learn (v0.24.2). The cohorts were split using an 80:20 ratio. Mann-Whitney *U* and *χ*^**2**^ tests (SciPy v1.6.3) were used to assess for significance in continuous and categorical clinical characteristics between the training and test cohorts respectively. A *p* value less than 0.05 was regarded as significant. Correlated features were removed if the Pearson coefficient was over 0.8. Seven different machine learning methods were used to create prediction models (scikit-learn v0.24.2): random forest, logistic regression (elastic net, lasso and ridge penalties explored), k-nearest neighbour (KNN), single-layer perceptron (SLP), multi-layer perceptron (MLP), Gaussian process classifier (GCP) and support vector machine (SVM). A maximum number of five features were selected for each of these models. The features selected in each method are based on the highest mean receiver operating characteristic (ROC) area under the curve (AUC) in five-fold stratified cross-validation with 20 repeats.

Each model was trained and tuned on the training cohort, using a five-fold cross-validation stratified around 2-EFS, again with 20 repeats. The model, hyperparameter and feature selection combination with the highest mean validation score from both the 4.0 SUV and 1.5 × mean liver segmentation were tested once on the unseen test cohort data. Given the growing literature surrounding the use of MTV as an outcome predictor, a separate logistic regression model using total MTV was trained in addition to a model using only clinical features and a combined clinical and MTV model. AUCs were compared using the DeLong method [20]. An appropriate threshold from the ROC curve for each of the best performing models was derived using the Youden index with the Matthews correlation coefficient (MCC), sensitivity, specificity, positive predictive value (PPV) and negative predictive value (PPV) presented.

## Results

### Patient demographics

A total of 289 patients were included in the study, with the patient demographics detailed in Table 2. There were no significant differences in the clinical characteristics between training and test cohorts.

**Table 2:** Demographics of the training and testing groups

	Training (*n* = 231)	Test (*n* = 58)	*p* value
**Age** (median)	36	41.5	0.10
**Sex**			
Male	124	29	0.72
Female	107	29	
**Ethnicity**			0.35
Caucasian	155	37	
Non-Caucasian	26	4	
Not disclosed	50	17	
**Stage**			0.13
1	14	5	
2	120	20	
3	46	17	
4	51	16	
**Chemotherapy**			0.11
ABVD/AVD	199	55	
Other	32	3	
**Radiotherapy**			0.87
No	179	45	
Yes	52	13	
**Treated as advanced disease**			0.55
No	59	12	
Yes	172	46	
**2-year EFS event**			0.99
No	177	45	
Yes	54	13	

*2-EFS*, 2-year event-free survival. The *p* values were calculated using a *t*-test for age and a *χ*^2^ test for the remaining demographic features

### Bin widths

For both the 4.0 SUV and 1.5 × mean liver SUV segmentation techniques, bin widths for PET and CT data were most robust when derived from the maximum range of SUV or HU respectively divided by 128 (Supplementary Figures 1 and 2). Overall, the 4.0 SUV segmentation technique resulted in more radiomic features being robust than the 1.5 × mean liver SUV segmentation method.

### Clinical- and MTV-derived models of 2-EFS

Patients who had a 2-EFS event had a significantly larger MTV compared to those who did not have a 2-EFS event. This was true for both segmentation techniques. With the 4.0 SUV method, the median MTVs were 167.4 cm^3^ versus 87.9 cm^3^ (*p* = 0.03); and for the 1.5 × mean liver SUV method, 324.3 cm^3^ versus 148.6 cm^3^ (*p* = 0.009). The median volumes were significantly greater in patients treated as advanced disease. For the 4.0 SUV method, the median MTVs were 250.6 cm^3^ (2-EFS event) versus 110.4 cm^3^ (no event) (*p* = 0.03); and for the 1.5 × mean liver SUV method, 457.8 cm^3^ (2-EFS event) versus 227.9 cm^3^ (no event) (*p* = 0.02)

A logistic regression model using MTV derived from a 4.0 SUV method resulted in a mean training AUC of 0.61 ± 0.02 (mean ± 95% CI) and a mean validation AUC of 0.61 ± 0.10 with the odds ratio being 1.00038 (Table 3). The logistic regression model derived from MTV using the 1.5 × mean liver SUV method had a mean training AUC of 0.63 ± 0.02 and a mean validation AUC of 0.63 ± 0.10, with the odds ratio being 1.00038.

**Table 3:** Mean training and validation scores for the best performing clinical- and metabolic tumour volume (MTV)–based logistic regression models

Model	Selected features	Hyperparameters	Mean train score (95% CI)	Mean validation score (95% CI)
Logistic regression – clinical	Cancer stage 1, cancer stage 4, age	C: 10, penalty: l2, Solver: newton-cg	0.74 ± 0.004	0.74 ± 0.02
Logistic regression – MTV (1.5 × mean liver SUV	MTV	C: 1e-07, penalty: l2, Solver: liblinear	0.63 ± 0.02	0.63 ± 0.10
Logistic regression – MTV (4.0 SUV)	MTV	C: 1e-07, penalty: l2, Solver: liblinear	0.62 ± 0.02	0.61 ± 0.10
Logistic regression – clinical and MTV (1.5 × mean liver SUV)	Cancer stage 1, Cancer stage 4, Age, MTV	C: 1, penalty: l2, Solver: saga	0.75 ± 0.004	0.74 ± 0.02

*l2*, Ridge regression penalty; *liblinear*, a library for large linear classification

**Fig. 1:**
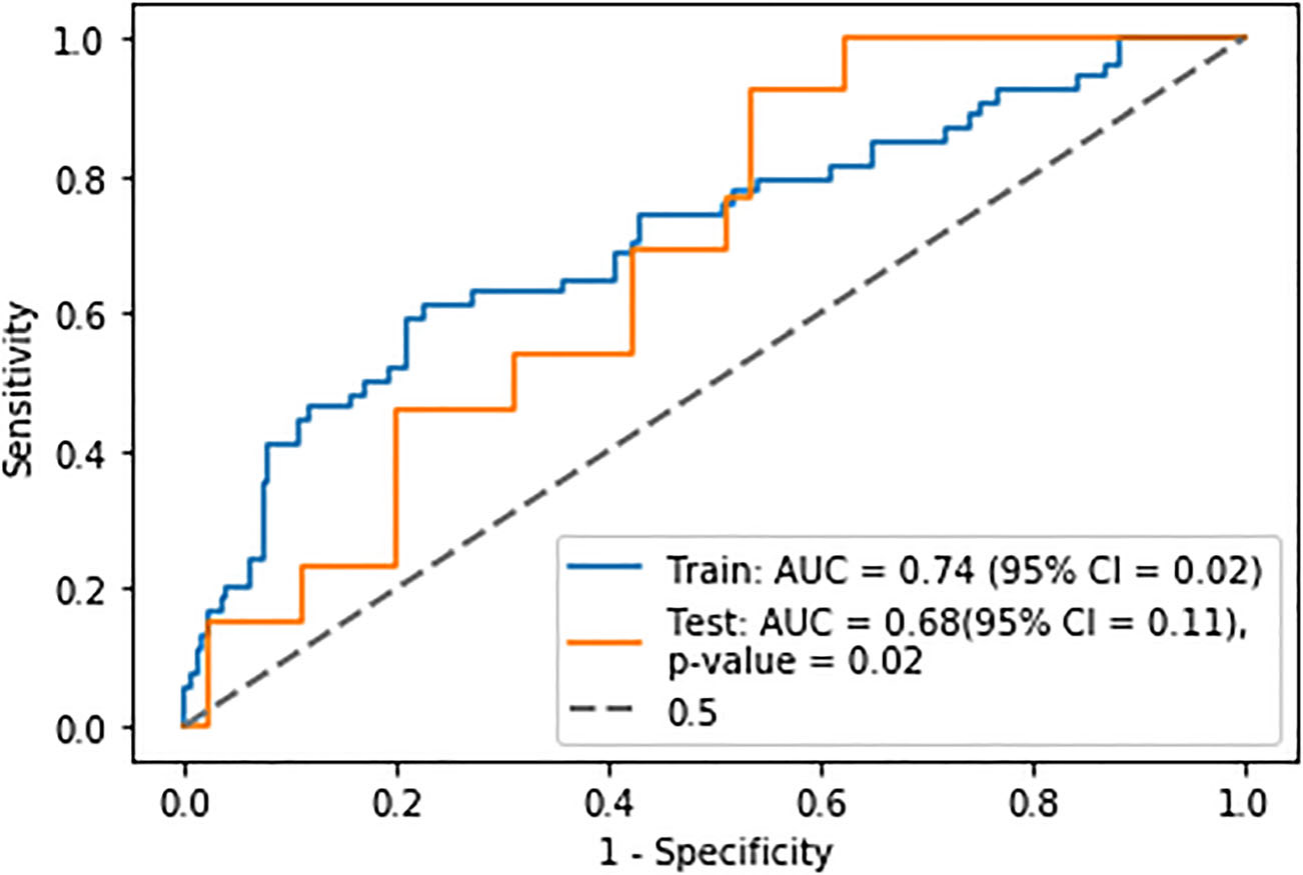
Receiver operator characteristic curve for the best performing predictive model derived from a logistic regression using MTV extracted from a 1.5 × mean liver SUV threshold segmentation technique and clinical features. The *p* value represents the comparison of the ROC to that of the 0.5 curve

Cancer stage 1, cancer stage 4 and age were selected as features for the clinical-based logistic regression model. This had a mean training AUC of 0.74 ± 0.004 and a mean validation AUC of 0.74 ± 0.02. When combing the features from this model with 1.5 × mean liver SUV MTV, the model had a mean training AUC of 0.74 ± 0.004 and a mean validation AUC of 0.72 ± 0.01. This model was tested on the unseen test set and achieved an AUC of 0.68 ± 0.11 (Fig. 1), MCC of 0.27, sensitivity of 0.31, specificity of 0.91, NPV of 0.47 and PPV of 0.85 at a threshold of 0.45.

### Clinical and radiomic model for the prediction of 2-EFS

**Fig. 2:**
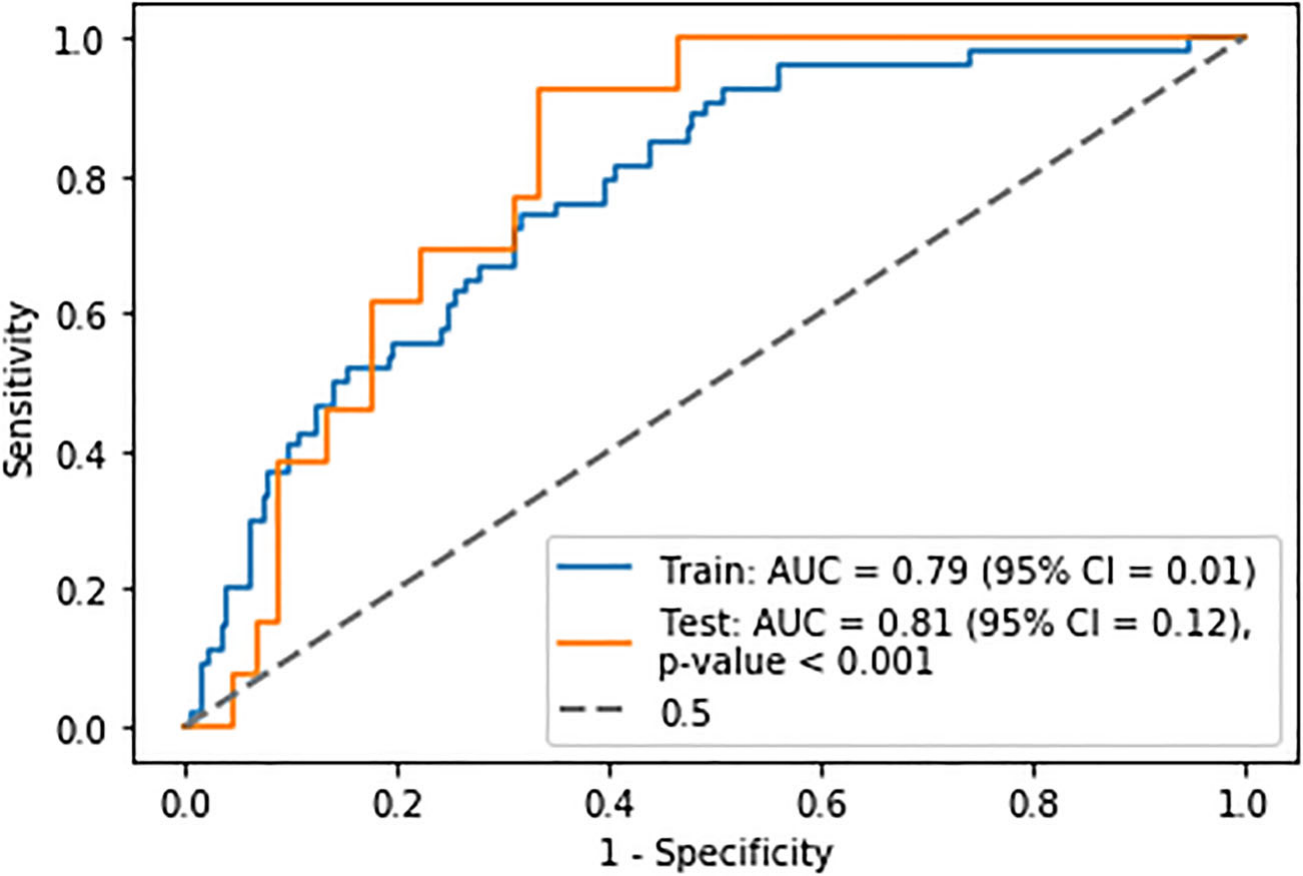
Receiver operator characteristic curve for the best performing predictive model derived from ridge regression using age and radiomic features extracted from a 1.5 × mean liver SUV threshold segmentation technique. The *p* value represents the comparison of the ROC to that of the 0.5 curve

The predictive model with the highest AUC was a ridge regression model derived from clinical and radiomic features extracted from the 1.5 × mean SUV threshold segmentation technique (Table 4). The model was constructed using features selected using a forward wrapper with five features chosen. The hyperparameters of the model were as follows: C = 1, penalty = l2 and solver = sag, class weight = balanced. The features chosen were age, PET flatness, PET major axis length, PET logarithm GLSZM size zone non-uniformity normalised, PET lbp-3D-m1 GLCM correlation and PET lbp-3D-m2 first order skewness. The mean training AUC was 0.82 ± 0.002, the mean validation AUC was 0.79 ± 0.01 and the test AUC was 0.81 ± 0.12 (Fig. 2), with MCC = 0.43, sensitivity = 0.42, specificity = 0.94, NPV = 0.67 and PPV = 0.85. The demographics of the mislabelled patients are presented in Supplementary Material 2: Table 2.

**Table 4:** Mean training and validation scores for the best performing machine learning models using a fixed threshold of 4.0SUV and 1.5 × mean liver SUV thresholding segmentation techniques

Model	Selected features	Hyperparameters	Mean train score (95% CI)	Mean validation score (95% CI)
**4.0 SUV**				
Support vector machine	Age, PET GLCM Imc1, PET wavelet-LLH GLCM Imc2, PET wavelet-HLL GLSZM small area emphasis, PET log-sigma-2-0-mm-3D GLSZM small area emphasis	C: 15.78, Gamma: 0.000794, Kernel: sigmoid	0.68 ± 0.004	0.66 ± 0.02
Logistic regression	Age, PET least axis length, PET wavelet-HLL GLCM correlation, PET wavelet-HLH GLCM Idmn, CT wavelet-HLL GLSZM large area low grey level emphasis	C: 1, penalty: l2, Solver: lbfgs	0.80 ± 0.002	0.78 ± 0.01
Random forest	Age	Bootstrap: true, Max depth: 1, min samples per leaf: 11, min samples per split: 32, number of estimators: 213	0.67 ± 0.004	0.64 ± 0.02
Multi-layer perceptron	Age, PET major axis length, PET wavelet-HHL GLCM Imc1, PET lbp-3D-k first order 10th percentile	Learning rate: invscaling, Solver: sgd	0.68 ± 0.004	0.68 ± 0.02
**1.5 × mean liver SUV**				
Support vector machine	PET first order 90th percentile, PET wavelet-LHH GLDM dependence non-uniformity normalised	C: 3.398, Gamma: 0.1005, Kernel: sigmoid	0.54 ± 0.008	0.55 ± 0.02
Logistic regression	Age, PET flatness, PET major axis length, PET logarithm GLSZM size zone non-uniformity normalised, PET lbp-3D-m1 GLCM correlation, PET lbp-3D-m2 first order skewness	C: 1, penalty: l2, Solver: sag	0.82 ± 0.002	0.79 ± 0.01
Random forest	Age	Bootstrap: true, Max depth: 1, min samples per leaf: 11, min samples per split: 48, number of estimators: 213	0.67 ± 0.004	0.64 ± 0.02
Multi-layer perceptron	Age, PET flatness, PET major axis length	Learning rate: invscaling, Solver: adam	0.77 ± 0.004	0.75 ± 0.01

The K-nearest neighbours, single-layer perceptron and Gaussian process classifier models were over-fitted with the mean training and validation AUCs with > 0.10 difference between the two. *l2*, Ridge regression penalty; *liblinear*, a library for large linear classification; *GLSZM*, grey level size zone matrix; *GLCM*, grey level co-occurrence matrix; *GLDM*, grey level dependence matrix; *rbf*, radial basis function; *L*, low; *H*, high; *Imc1*, informational measure of correlation 1; *Imc2*, informational measure of correlation 2; *idmn*, inverse difference moment normalised; *lbp*, local binary pattern

**Fig. 3:**
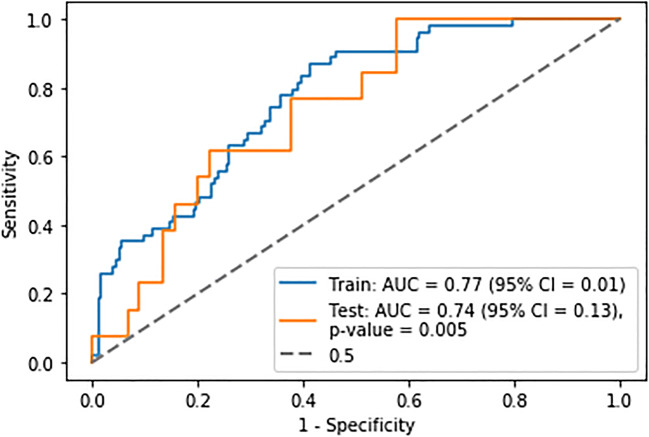
Receiver operator characteristic curve for the best performing predictive model derived from ridge regression using age and radiomic features extracted using a 4.0 SUV fixed threshold segmentation technique. The *p* value represents the comparison of the ROC to that of the 0.5 curve

The highest performing predictive model using the 4.0 SUV threshold was a regression model using a ridge regression penalty with a mean training AUC of 0.79 ± 0.002, the mean validation AUC of 0.77 ± 0.01 and the test AUC of 0.74 ± 0.13 (Fig. 3). The MCC = 0.30, sensitivity = 032, specificity = 0.95, NPV = 0.42 and PPV = 0.92 at a threshold of 0.27. The model was constructed using features selected from a forward wrapper method of feature selection with five features chosen. The hyperparameters of the model were as follows: C = 100, penalty = l2 and solver = saga, class weight = balanced.

**Fig. 4:**
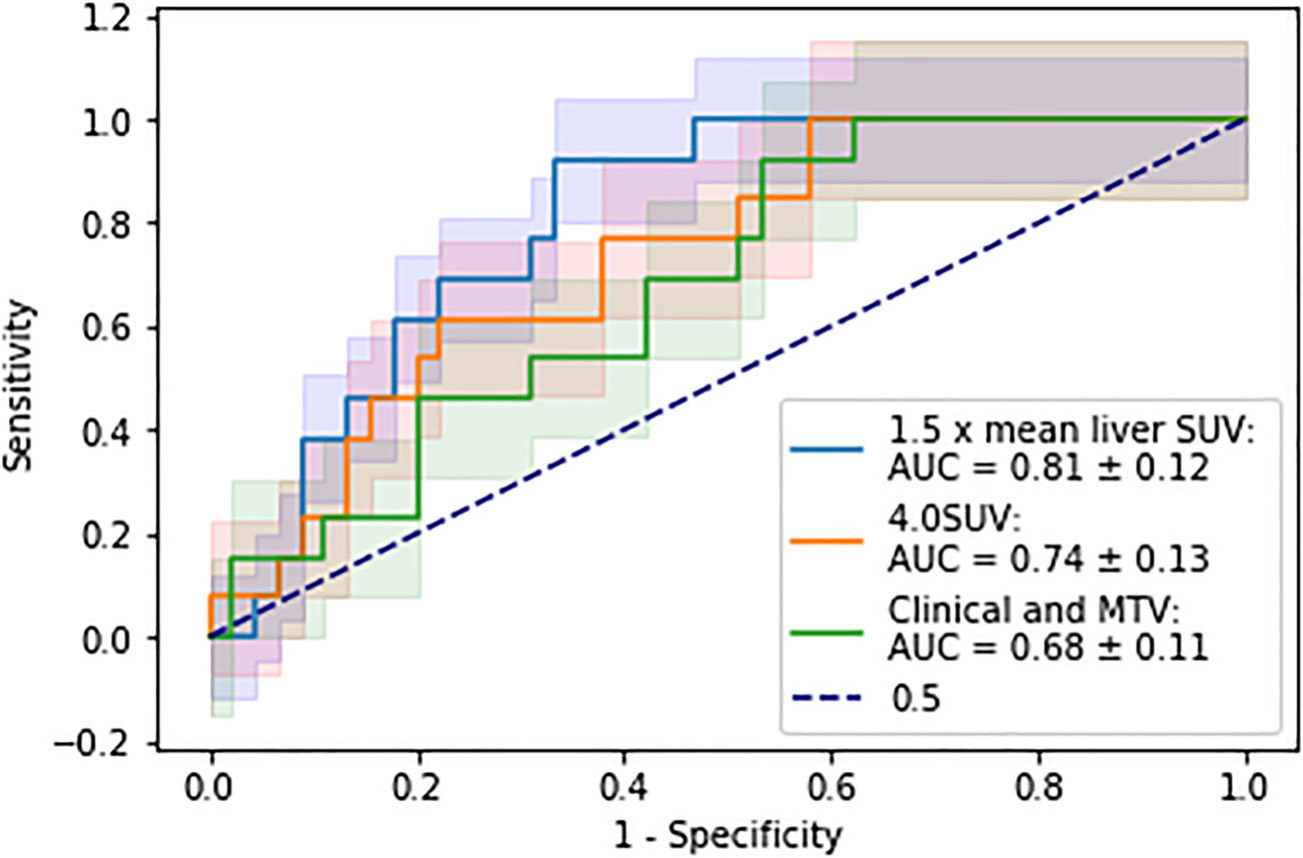
Receiver operator curves, with associated confidence intervals, for the best performing MTV and radiomic models derived from 4.0 SUV fixed threshold and 1.5 × mean liver SUV threshold segmentation techniques

There was no significant difference between the test set AUCs of the best performing clinical- and radiomic-based models with each other and with the best performing clinical- and MTV-based model (Fig. 4; Table 5). The intercept and coefficients for each model are presented in Supplementary Material 2: Table 3.

**Table 5:** Comparison of the different test AUCs using the DeLong method, *p* values presented

	Clinical and MTV	1.5 × mean liver SUV	4.0 SUV
**Clinical and MTV**	n/a	0.11	0.53
**1.5 × mean liver SUV**	0.11	n/a	0.22
**4.0 SUV**	0.53	0.22	n/a

## Discussion

This study confirms that pre-treatment outcome prediction using FDG PET/CT–derived radiomic features is feasible in patients with cHL. The best performing model was created using ridge regression combining age and four radiomic features (PET flatness, PET major axis length, PET logarithm GLSZM size zone non-uniformity normalised, PET lbp-3D-m1 GLCM correlation and PET lbp-3D-m2 first order skewness) extracted from PET images using a 1.5 × mean liver SUV method with a bin width of 0.24. It must be noted that there was no significant difference between the test AUC of this model and those of a combined clinical and MTV model and a model created using 4.0 SUV fixed threshold segmentation. This is likely due to small numbers involved given the relatively large confidence intervals. Due to missing clinical data, it was not possible to adjust for features used to stratify patients into early and advanced disease. A surrogate, treatment intent, was used instead which demonstrated that the models created remained reasonable predictors of outcome for patients treated as having advanced disease.

Further work should be performed to assess the relationship of ethnicity and socio-economic status on a model’s predictive ability to avoid creation of a model which discriminates against under-represented subsets of patients due to lack of data to train and test the model on [21, 22]. Unadjusted confounders are likely one of the reasons for a minority of studies reporting the poor ability of MTV as an outcome predictor in lymphoma [23–25]. Most notably Adams et al found that MTV was not an independent predictor of overall survival or PFS in diffuse large B cell lymphoma once adjusting for the National Comprehensive Cancer Network International Prognostic Index [26]. To allow for transparency, our study has provided the demographic information for the patients who were mislabelled using the predictive model with the highest test AUC.

**Fig. 5:**
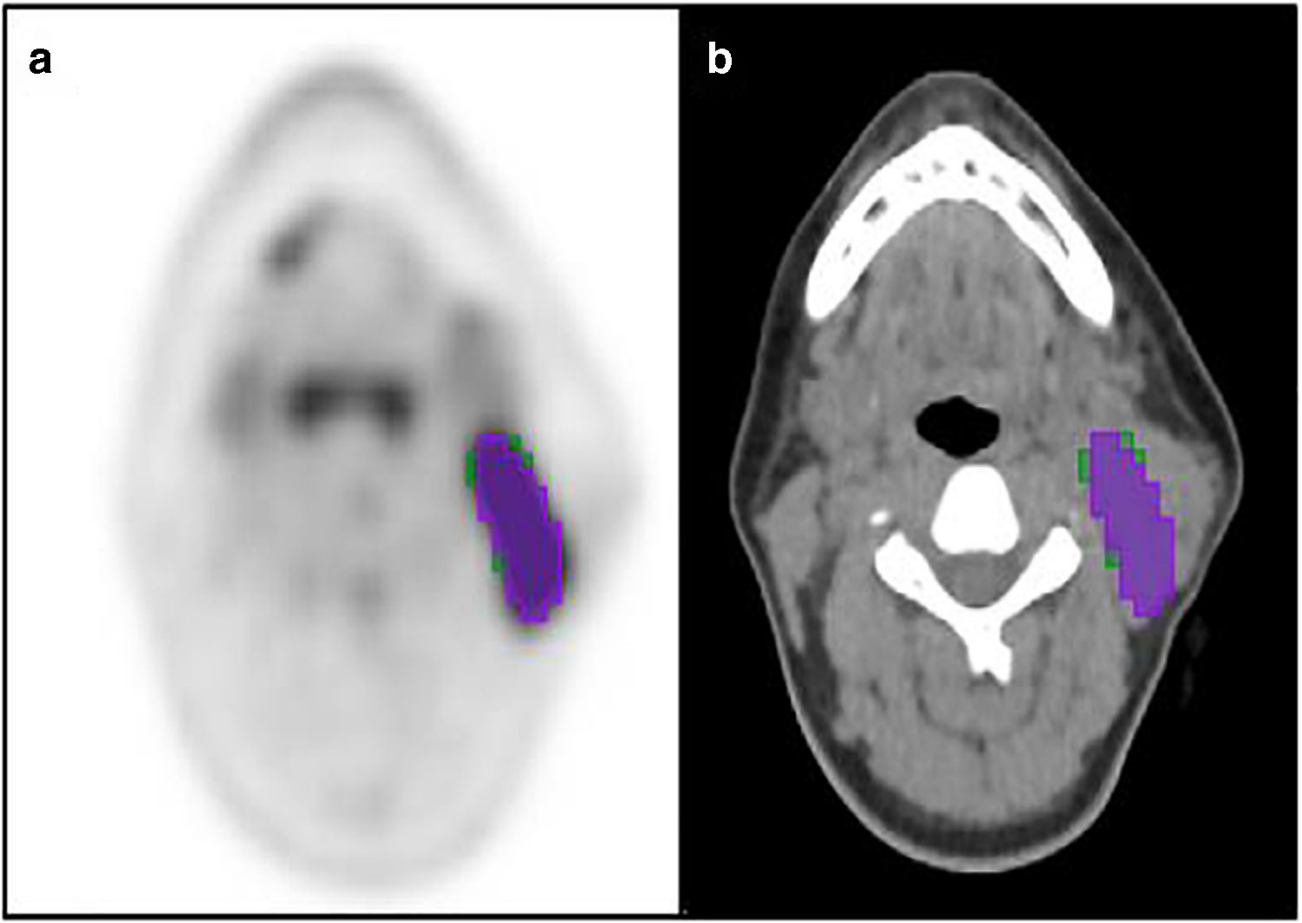
Select axial slice through PET (**a**) and CT (**b**) images of a patient with Hodgkin lymphoma demonstrating a pathological left level II lymph node. The purple segmentation represents the original 4.0 SUV fixed threshold segmentation performed using Mirada Medical RTx (v1.8.2) software and the green areas represent the additional area included when segmented with a fixed 4.0 SUV threshold using 3D Slicer (v4.11) software

Two different segmentation techniques were explored. The first was a fixed threshold of 4.0 SUV which has been demonstrated to be a reproducible, efficient method for contouring disease [27]. The second was 1.5 × mean liver SUV which has been explored in other malignancies and provides an adaptive threshold which adjusts for background SUV uptake [17, 18]. Our study echoed previous work demonstrating a fixed threshold led to more features being robust following re-segmentation. The fixed thresholding segmentation technique required less steps, and less manual adaption [28]. However, a fixed SUV thresholding technique does not scale with the physiological uptake and therefore, the contours may vary on repeat studies due to external effects on the SUV rather than tumour pathophysiology [27]. The study also demonstrated the variability which can occur when repeating a segmentation methodology on different software (Fig. 5), with radiomic features not being deemed robust following repeated segmentation even when using the same SUV thresholds. ComBat harmonisation was employed to mitigate against the effects of scanner variation. This is based on Bayes theorem and attempts to predict scanner influence whilst maintaining biological variation [29]. For this to be effective, however, there must be enough samples from different scanners to apply the harmonisation method [30] and it cannot be applied prospectively to scanner acquisitions outside those used for training of the predictive model.

Previous studies have explored the use of radiomic features in the prediction of outcomes in HL [13–15]. Lue et al found SUV kurtosis, stage and intensity non-uniformity (INU) derived from Grey Level Run Length Matrix (GLRLM) were independent predictors of PFS in a small cohort of 42 patients. Milogrom et al demonstrated that the combination of SUVmax, MTV, InformationMeasureCorr1, InformationMeasureCorr2 and InverseVariance derived from GLCM 2.5 had an AUC of 0.95 when predicting relapse in 167 patients with stage I–II HL. However, there were very few events, with the validation cohort only having two patients who relapsed. Sollini et al assessed a radiomic fingerprint using principal component analysis to classify patients who would relapse within 4 years of treatment in a cohort of 85 patients. They explored fingerprints created from a single largest nodal or extra-nodal lesion versus using all lesions and found that the intra-patient similarity was low, and that the highest accuracy was achieved when using all lesions within the model [15]. This highlights the inherent heterogeneity of radiomic features within different lesions and that by restricting analysis to a single lesion, the predictive model may also be limited. The current study of 289 patients is one of the largest to assess potential utility of radiomic features derived from pre-treatment FDG PET/CT for predicting outcome in cHL patients. It demonstrates that radiomics could feasibly improve prediction of 2-EFS. However, this requires validation on an independent external dataset and although the AUC for the test set was 0.81, no clear predictive threshold could be derived. This must be a key target when creating any machine learning or AI-based model. In terms of HL, it would be the ability to balance side effects of escalated treatment, with the rates of EFS and toxicity varying between treatment regimens [31]. The advent of newer therapeutic strategies limits the use of predictive models made on retrospective data; future efforts should focus on validating imaging, genetic and clinical predictive features in carefully designed prospective, multi-centre clinical trials.

A TRIPOD checklist was used to ensure transparency of the study’s methodology, a concern in previous radiomic studies [32, 33]. However, no external validation was performed, and although contouring was undertaken without knowledge of clinical outcome, no measures to blind assessors were specifically undertaken. Although patients with other concurrent malignancies were excluded from analysis, other pathologies were not taken into consideration when looking at mortality. Other study limitations include its retrospective nature, the relatively small event rate, reliance on clinical records to determine date of relapse/recurrence, exclusion of patients with hepatic disease/or without disease > 4.0 SUV and variation in different patient’s treatment regimen.

## Conclusion

There is potential for models derived from radiomic features extracted from pre-treatment FDG PET/CT to predict 2-EFS in cHL patients. Further work is needed to determine optimum thresholds for clinical use.

## Supplementary information

ESM 1 (PDF 540 kb)

## Abbreviations

ABVD: Doxorubicin (Adriamycin), bleomycin, vinblastine and dacarbazine
AUC: Area under the curve
BEACOPP: Bleomycin, etoposide, doxorubicin (Adriamycin), cyclophosphamide, vincristine (Oncovin), procarbazine, and prednisone
cHL: Classical Hodgkin lymphoma
CMR: Complete metabolic response
ComBat: Combating batch effects when combining batches
DICOM: Digital imaging and communications in medicine
EFS: Event-free survival
FDG: 2-deoxy-2-[^18^F]fluoro-d-glucose
GLCM: Grey level co-occurrence matrix
GLDM: Grey level dependence matrix
GLRLM: Grey level run length matrix
GLSZM: Grey level size zone matrix
HL: Hodgkin lymphoma
HU: Hounsfield units
Id: Inverse difference
Idm: Inverse difference moment
Idmn: Inverse difference moment normalised
Idn: Inverse difference normalised
Imc: Informational measure of correlation
KNN: k-nearest neighbour
lbfgs: Limited memory Broyden-Fletcher-Goldfarb-Shanno
lbp: Local binary pattern
LGLE: Low grey level emphasis selected
MCC: Matthews correlation coefficient
MTV: Metabolic tumour volume
NGTDM: Neighbouring grey tone difference matrix
NIfTI: Neuroimaging Informatics Technology Initiative
NLPHL: Nodular lymphocyte-predominant HL
NPV: Negative predictive value
PET/CT: Positron emission tomography/computed tomography
PPV: Positive predictive value
ROC: Receiver operating characteristic
ROI: Region of interest
RT: Radiotherapy
SUV: Standardised uptake value
SVM: Support vector machine
TLG: Total lesion glycolysis
TRIPOD: Transparent reporting of a multivariable prediction model for individual prognosis or diagnosis

## Declarations

### Guarantor

The scientific guarantor of this publication is Prof. Andrew Scarsbrook.

### Statistics and biometry

No complex statistical methods were necessary for this paper.

### Methodology

• retrospective

• diagnostic or prognostic study

• performed at one institution
