# Supplementary material for: Utility of pre-treatment FDG PET/CT–derived machine learning models for outcome prediction in classical Hodgkin lymphoma
Source: Eur Radiol. 2022 Aug 25;32(10):7237–47. doi: 10.1007/s00330-022-09039-0 (PMC9403224; doi:10.1007/s00330-022-09039-0)

Section/Topic		Checklist Item	Page
**Title and abstract**			
Title	1	Identify the study as developing and/or validating a multivariable prediction model, the target population, and the outcome to be predicted.	*
Abstract	2	Provide a summary of objectives, study design, setting, participants, sample size, predictors, outcome, statistical analysis, results, and conclusions.	*
**Introduction**			
Background and objectives	3a	Explain the medical context (including whether diagnostic or prognostic) and rationale for developing or validating the multivariable prediction model, including references to existing models.	*
	3b	Specify the objectives, including whether the study describes the development or validation of the model or both.	*
**Methods**			
Source of data	4a	Describe the study design or source of data (e.g., randomized trial, cohort, or registry data), separately for the development and validation data sets, if applicable.	*
	4b	Specify the key study dates, including start of accrual; end of accrual; and, if applicable, end of follow-up.	*
Participants	5a	Specify key elements of the study setting (e.g., primary care, secondary care, general population) including number and location of centres.	*
	5b	Describe eligibility criteria for participants.	*
	5c	Give details of treatments received, if relevant.	*
Outcome	6a	Clearly define the outcome that is predicted by the prediction model, including how and when assessed.	*
	6b	Report any actions to blind assessment of the outcome to be predicted.	*
Predictors	7a	Clearly define all predictors used in developing or validating the multivariable prediction model, including how and when they were measured.	*
	7b	Report any actions to blind assessment of predictors for the outcome and other predictors.	*
Sample size	8	Explain how the study size was arrived at.	*
Missing data	9	Describe how missing data were handled (e.g., complete-case analysis, single imputation, multiple imputation) with details of any imputation method.	*
Statistical analysis methods	10a	Describe how predictors were handled in the analyses.	*
	10b	Specify type of model, all model-building procedures (including any predictor selection), and method for internal validation.	*
	10d	Specify all measures used to assess model performance and, if relevant, to compare multiple models.	*
Risk groups	11	Provide details on how risk groups were created, if done.	N/A
**Results**			
Participants	13a	Describe the flow of participants through the study, including the number of participants with and without the outcome and, if applicable, a summary of the follow-up time. A diagram may be helpful.	*
	13b	Describe the characteristics of the participants (basic demographics, clinical features, available predictors), including the number of participants with missing data for predictors and outcome.	*
Model development	14a	Specify the number of participants and outcome events in each analysis.	*
	14b	If done, report the unadjusted association between each candidate predictor and outcome.	N/A
Model specification	15a	Present the full prediction model to allow predictions for individuals (i.e., all regression coefficients, and model intercept or baseline survival at a given time point).	*
	15b	Explain how to the use the prediction model.	*
Model performance	16	Report performance measures (with CIs) for the prediction model.	Fig 4
**Discussion**			
Limitations	18	Discuss any limitations of the study (such as nonrepresentative sample, few events per predictor, missing data).	*
Interpretation	19b	Give an overall interpretation of the results, considering objectives, limitations, and results from similar studies, and other relevant evidence.	*
Implications	20	Discuss the potential clinical use of the model and implications for future research.	*
**Other information**			

Supplementary information	21	Provide information about the availability of supplementary resources, such as study protocol, Web calculator, and data sets.	*
Funding	22	Give the source of funding and the role of the funders for the present study.	N/A

*See comments

We recommend using the TRIPOD Checklist in conjunction with the TRIPOD Explanation and Elaboration document.

This study looks at the utility of pre-treatment FDG PET/CT derived machine learning models for outcome prediction in classical Hodgkin lymphoma. (Title)

The abstract covers a summary of all the requested information. (Abstract)

a) The introduction presents the background of HL sets out the aim of creating a predictive model using machine learning techniques using radiomic features derived from the baseline PET/CT. Two previous papers are discussed which aim to create a similar model. (Introduction)
b) The aim of this study was to create a predictive model using radiomic features derived from pre-treatment FDG PET/CT to predict 2-year EFS in HL patients using a larger tertiary centre cohort of patients (Introduction)

a) This is a retrospective single centre cohort study. The study cohort was randomised on a ratio of 4:1 into training and testing cohorts stratified around 2-EFS, age, gender, ethnicity and disease stage. (Patient selection)

b) Consecutive patients with histologically proven cHL who underwent baseline FDG-PET/CT at a single large tertiary referral centre between June 2008 and January 2018 were included. The follow up information recorded is set out in the patient selection section. (Patient selection)

a) This is a single tertiary centre study. (Patient selection)
b) Patients were excluded if they were under 16 years of age, did not have cHL, had treatment prior to their staging PET/CT study, did not have measurable disease on PET/CT, had a concurrent malignancy, they did not have disease over 4.0SUV, had hepatic involvement or if the images were degraded or incomplete. The follow up information recorded is set out (Patient selection)
c) The treatment regimen for the cohort is set out in Table 2. No change to departmental standard treatment was performed. (Table 2)

a) An event was defined as relapse, recurrence or death within the 2 year follow up period. (Patient selection)
b) As this was a retrospective study the primary outcomes were defined from clinical records. The investigator reviewing the records was blinded to the imaging parameters.

a) The description of the contouring method, resampling, harmonisation, radiomic feature extraction and the methods used for feature selection are documented within the method section and Supplementary Material 2. The features selected as part of the models are described in Table 3. (Materials and methods, Supplementary Material 2, Results)
b) The images were contoured and analysed without reference to the outcome data.

All patients which met the inclusion criteria were included. The cut-off of January 2018 was chosen to allow for 2 year follow up without confounding factors introduced due to the covid-19 pandemic. For feature selection 5 features were chosen as the maximum number of features to be include in each model. This was derived from 10 events per parameter, with 54 events within the training cohort. (**Materials and methods, Results**)

Only complete data sets were used in the analysis. (**Results**)

. a) Clinical factors were included in the variable selection process alongside radiomic features. The categorical data was dummy encoded. Continuous features were normalised using a standard scaler. (**Machine learning analysis, Supplementary Material 2**)
b) Random forest, support vector machine, logistic regression, k-nearest neighbour, single layer perceptron, multi-layer perceptron and Gaussian process classifier models were trained and tuned on the training cohort using cross validation. The models were created using different feature selection methods, the bin width or bin number was selected based on the method which had the greatest robust features (intraclass correlation coefficient >0.8) following regimentation. A model was generated using radiomic features from a fixed 4.0 SUV threshold segmentation technique and a 1.5 x mean liver SUV threshold segmentation technique. A model was also created using metabolic tumour volume. The models with the highest mean receiver operating characteristic (ROC) area under the curve (AUC) were tested and compared on the unseen test cohort. (**Machine learning analysis, Supplementary Material 2**,)
d) When comparing models, the mean validation AUC was used to determine the best performing model. A Delong test was used to compare the AUCs of the test set. (**Machine learning analysis, Supplementary Material 2**,)

. Risk groups were not created within the model.

. a) 289 patients were included, with demographics detailed in **Table 2.** (**Results**)
b) The characteristics of the participants are presented in **Table 2**.

. a) The number of events per cohort are presented in **Table 2**.
b) This has not been performed. The training and testing cohorts were stratified around key clinical features, but the results are not adjusted for these. Further analysis was performed looking at how the model performed on patients treated as having advanced disease.

. a/b) The features and hyperparameters used to create the model are presented in the **Clinical and radiomic model for the prediction of 2-EFS** section.

. The mean validation and test ROC curves are presented. The 95% confidence intervals are presented. (**Results**)

. The limitations of the study are presented. These include the retrospective nature of the study, the relative low number of events, reliance on clinical records, the exclusion of patients with hepatic disease or disease not meeting the 4.0 SUV threshold, variation in patient treatment and that there was no external validation. (**Discussion**)

. b)/20. The discussion section gives an overall interpretation of the results and highlights the potential use of a pre-treatment model to aid in early personalised treatment for patients. (Discussion)

. The python libraries used are references within the text. The radiomic features extracted using PyRadiomics are detailed in Supplementary Material Table 2.

. The study was not externally funded. Individual author’s funding is declared within the Declaration.

Supplementary Material 2

Image segmentation

Image data were viewed and contoured using specialised multimodality imaging software (RTx v1.8.2, Mirada Medical). Lymphomatous disease segmentation was performed by a clinical radiologist with six years' experience and a research radiographer with 2 years' experience of segmenting cross-sectional imaging and reviewed by two dual-certified Radiology and Nuclear Medicine Physicians with >15 years' experience of oncological PET/CT interpretation. Any discrepancies were agreed in consensus. Two segmentation techniques were utilised, the first using a fixed threshold of 4.0 SUV and the second using a threshold of 1.5 x liver SUVmean was used to contour disease sites on PET, this method has been used in different cancer types [16, 17]. The mean liver SUV was determined by placing a 110 cm^3 region of interest in the right lobe of the liver. The contour from the PET was translated to the co-registered unenhanced low-dose CT component of the study with the contours matched to soft tissue with a value of -10 to 100 Hounsfield units (HU). Contours were exported as digital imaging and communications in medicine (DICOM) radiotherapy (RT) structures. Ten percent of the cases were re-segmented using the same methodology described by the radiologist who performed the initial segmentation after a 3-month washout period using Slicer (v4.11). These segmentations were used to test the repeatability of the segmentation techniques and to test the robustness of the extracted features.

Feature extraction

DICOM images and DICOM-RT structures were converted to Neuroimaging Informatics Technology Initiative (NIfTI) files using the python library Simple ITK (v2.0.2). Absolute PET voxel values were converted to body weight SUV and voxel values for CT were converted to HU using the equations detailed below.

$$SUVbw = \frac{\left(\text{pixel value }\left(\frac{\text{Bq}}{\text{ml}}\right) \times \text{slope} + \text{intercept}\right) \times body\ weight\ (g)}{\text{tracer activity (Bq)} \times 2^{\left(-\left(\frac{scan\ time\ (s) - measured\ time\ (s)}{\text{half life (s)}}\right)\right)}}$$

$$HU = pixel\ value\ \times slope + intercept$$

Both CT and PET data were resampled to a uniform voxel size of 2 mm^3. The robustness of radiomic features to re-segmentation using different software was used to identify the optimum bin width for the dataset. Radiomic features were extracted using a fixed bin number of 32, 64 and 128, and bin widths derived from either dividing the maximum or median voxel range by 32, 64 and 128. Features were deemed to be robust if the intraclass correlation coefficient (ICC) calculated using the python library pingouin (v0.3.12) was >0.8. First and second order parameters were extracted using PyRadiomics (v2.2.0). There are some deviations between PyRadiomics and the image biomarker standardisation initiative (IBSI), with Pyradiomics starting the fixed bin width from 0 and not the minimum segmentation value, and the calculation of first order kurtosis being +3 larger in PyRadiomics [18, 19]. Patient age, histology and sex were also included as clinical features in the models. Disease stage and sex were dummy encoded using (Pandas v1.2.4). This resulted in a total of 3935 features extracted per segmentation technique for each patient (**Supplementary Table 1**). Harmonisation to account for the different scanners was applied to the radiomic features using the ComBat method (https://github.com/Jfortin1/ComBatHarmonization) [20].

Machine learning analysis

The study cohort was split into training and test cohorts stratified around 2-year EFS (2-EFS), age, sex, ethnicity, stage of disease, having radiotherapy, having ABVD-based chemotherapy and being treated as advanced disease using scikit-learn (v0.24.2). Ethnicity was defined by the volunteered information from patients. Given the low numbers of some ethnic groups, it was not possible to stratify the training and tests around ethnicity without splitting the data into Caucasian and non-Caucasian ethnic groups. The cohorts were split using an 80:20 ratio. Mann-Whitney U and χ^2 tests (SciPy v1.6.3) were used to assess for significance in continuous and categorical clinical characteristics between the training and test cohorts respectively. A p-value less than 0.05 was regarded as significant. Categorical data was dummy encoded (Pandas v1.2.4), and continuous data was normalised using a standard scaler (scikit-learn v0.24.2). Correlated features were removed if the Pearson coefficient was over 0.8. Seven different machine learning methods were used to create prediction models (scikit-learn v0.24.2): random forest, logistic regression (elastic net, lasso and ridge penalties explored), k-nearest neighbour (KNN), single layer perceptron (SLP), multi-layer perceptron (MLP), Gaussian process classifier (GCP) and support vector machine (SVM). A maximum number of five features was selected for the model and this was based on one feature per 10 events. Three feature selection methods were used: a forward wrapper method (mlxtend 0.18.0), a univariate analysis method (scikit-learn v0.24.2), and a recursive feature extraction method (for the models where this was applicable i.e. random forest and

logistic regression) (scikit-learn v0.24.2). For each of these methods, two to five selected features were evaluated in the machine learning models. The features selected in each method are based on the highest mean receiver operating characteristic (ROC) area under the curve (AUC) in five-fold stratified cross validation with 20 repeats.

Each model was then trained and tuned on the training cohort, using a stratified five-fold cross validation stratified around 2-EFS, again with 20 repeats. Hyperparameters were initially tuned using a random search cross validation with 1000 different combinations explored (scikit-learn v0.24.2). For all models the random state hyperparameter was set to a value of 0, and, where applicable, the class weight hyperparameter was set to “balanced” to help mitigate the unbalanced nature of the data. The hyperparameters of the 10 top highest validation scores from the random search cross validation were further explored using grid search cross validation (scikit-learn v0.24.2). For the combination of hyperparameters explored in the tuning process, if the mean training and mean validation AUC were not within 0.03 the model was discarded. The remaining models were ranked by the highest mean validation score. The model, hyperparameter and feature selection combination with the highest mean validation score from both the 4.0 SUV threshold segmentation and the 1.5 x mean liver SUV threshold were tested once on the unseen test cohort data. Given the growing literature surrounding the use of MTV as an outcome predictor a separate logistic regression model using MTV was trained on the training set and tested on the unseen test cohort as was used as a comparison to the best performing model. AUCs were compared using the DeLong method. An appropriate threshold from the ROC curve for each of the best performing models was derived using the Youden index with the Matthews correlation coefficient (MCC), sensitivity, specificity, positive predictive value (PPV) and negative predictive value (PPV) presented.

Missing clinical data meant that a comparison with commonly utilised clinical scoring methods was not possible and the treatment regime used was used a surrogate indicator of whether the patient was deemed to have early or advanced disease.

First Order	Shape	GLCM	GLRLM	GLDM	GLSZM	NGTDM
10th Percentile	Elongation	Autocorrelation	Grey Level Non-Uniformity	Dependence Entropy	Grey Level Non-Uniformity	Busyness
90th Percentile	Flatness	Cluster Prominence	Grey Level Non-Uniformity Normalized	Dependence Non-Uniformity	Grey Level Non-Uniformity Normalized	Coarseness
Energy	Least Axis Length	Cluster Shade	Grey Level Variance	Dependence Non-Uniformity Normalized	Grey Level Variance	Complexity
Entropy	Major Axis Length	Cluster Tendency	High Grey Level Run Emphasis	Dependence Variance	High Grey Level Zone Emphasis	Contrast
Inter quartile Range	Maximum 2D Diameter Column	Contrast	Long Run Emphasis	Grey Level Non-Uniformity	Large Area Emphasis	Strength 5
Kurtosis	Maximum 2D Diameter Row	Correlation	Long Run High Grey Level Emphasis	Grey Level Variance	Large Area High Grey Level Emphasis	
Maximum	Maximum 2D Diameter Slice	Difference Average	Long Run Low Grey Level Emphasis	High Grey Level Emphasis	Large Area Low Grey Level Emphasis	
Mean Absolute Deviation	Maximum 3D Diameter	Difference Entropy	Low Grey Level Run Emphasis	Large Dependence Emphasis	Low Grey Level Zone Emphasis	
Mean	Mesh Volume	Difference Variance	Run Entropy	Large Dependence High Grey Level Emphasis	Size Zone Non-Uniformity	
Median	Minor Axis Length	Id	Run Length Non-Uniformity	Large Dependence Low Grey Level Emphasis	Size Zone Non-Uniformity Normalized	
Minimum	Sphericity	Idm	Run Length Non-Uniformity Normalised	Low Grey Level Emphasis	Small Area Emphasis	
Range	Surface Area	Idmn	Run Percentage	Small Dependence Emphasis	Small Area High Grey Level Emphasis	

Robust Mean Absolute Deviation	Surface Volume Ratio	Idn	Run Variance	Small Dependence High Grey Level Emphasis	Small Area Low Grey Level Emphasis	
Root Mean Squared	Voxel Volume 14	Imc1	Short Run Emphasis	Small Dependence Low Grey Level Emphasis	Zone Entropy	
Skewness	15	Imc2	Short Run High Grey Level Emphasis		Zone Percentage	
Total Energy	16	Inverse Variance	Short Run Low Grey Level Emphasis		Zone Variance	
Uniformity	17	Joint Average				
Variance 18	18	Joint Energy				
	19	Joint Entropy				
	20	MCC				
	21	Maximum Probability				
	22	Sum Average				
	23	Sum Entropy				
	24	Sum Squares				

Supplementary Table 1: detailing the radiomic features extracted for both the PET and CT components. The equations for the features can be found at https://pyradiomics.readthedocs.io/en/latest/features.html. GLCM = grey level co-occurrence matrix, GLDM = grey level dependence matrix, GLRLM = grey level run length matrix, GLSZM = grey level size zone matrix, NGTDM = neighbouring grey tone difference matrix, Id = inverse difference, Idn = inverse difference normalised, Imc = informational measure of correlation, Idm = inverse difference moment, Idmn = inverse difference moment normalised, MCC = Matthews correlation coefficient. Each of the first and second order features were extracted from the original imaging and then from the images following filters applied. The filters used were: wavelet (LLL, LLH, LHL, LHH, HHH, HLH, HHL, HLL); log-signa (1.0, 2.0, 3.0, 4.0); square; square root; logarithm; exponential; gradient; lbp-3D (m1, m2, k).

2-year EFS: Prediction	2-year EFS: True	Age Group	Sex	Ethnicity	Cancer Stage	Treated as advanced disease	Radiotherapy
0	1	60-69	Female	Caucasian	3	1	0
1	0	70-79	Male	Caucasian	3	1	1
1	0	60-69	Female	Caucasian	4	1	0
1	0	40-49	Female	Caucasian	3	1	1
1	0	40-49	Female	Caucasian	2	0	1
1	0	80-89	Male	Caucasian	2	0	1
1	0	70-79	Male	Caucasian	2	1	0
1	0	30-39	Female	Caucasian	2	1	0
1	0	60-69	Male	Caucasian	3	1	0
1	0	70-79	Female	Caucasian	2	0	1
1	0	40-49	Male	Caucasian	2	1	1
1	0	50-59	Male	Caucasian	3	1	0
1	0	20-29	Male	Non-Caucasian	3	1	0
0	1	40-49	Male	Caucasian	4	1	0
1	0	40-49	Male	Caucasian	4	1	0
1	0	70-79	Male	Not given	4	1	0
1	0	70-79	Female	Not given	3	1	1

Supplementary Table 2: Patient information for mislabelled test cases when using the 1.5 x mean liver SUV combined clinical and radiomic ridge regression model.

Model	Intercept	Coefficients
Clinical and MTV	-0.35815567	Cancer stage 1: 5.02009465 , Cancer stage 4: -1.27629249, Age: 0.4807701, MTV: 0.15398729
1.5 x mean liver SUV	-0.42846688	Age: 0.86012792, PET flatness: 0.75497062, PET major axis length: 1.05538773, PET logarithm GLSZM size zone non-uniformity normalized: -0.57813534, PET lbp-3D-m1 GLCM correlation: 0.61007467, PET lbp-3D-m2 first order skewness: -0.84823908
4.0 SUV	-0.41354898	Age: 0.73897899, PET least axis length: 1.10580035, PET wavelet-HLL GLCM correlation: -0.75524818, PET wavelet-HLH GLCM Idmn: -0.488136, CT wavelet-HLL GLSZM large area low gray level emphasis: -0.85812909

Supplementary Table 2: Intercept and coefficients for the best performing clinical and MTV, and radiomic logistic regression models. GLSZM = grey level size zone matrix, GLCM = grey level co-occurrence matrix, GLDM = grey level dependence matrix, rbf = radial basis function, L = low, H = high, Imc1 = informational measure of correlation 1, Imc2 = informational measure of correlation 2, idmn = inverse difference moment normalized, lbp = local binary pattern.

Supplementary Figure 1

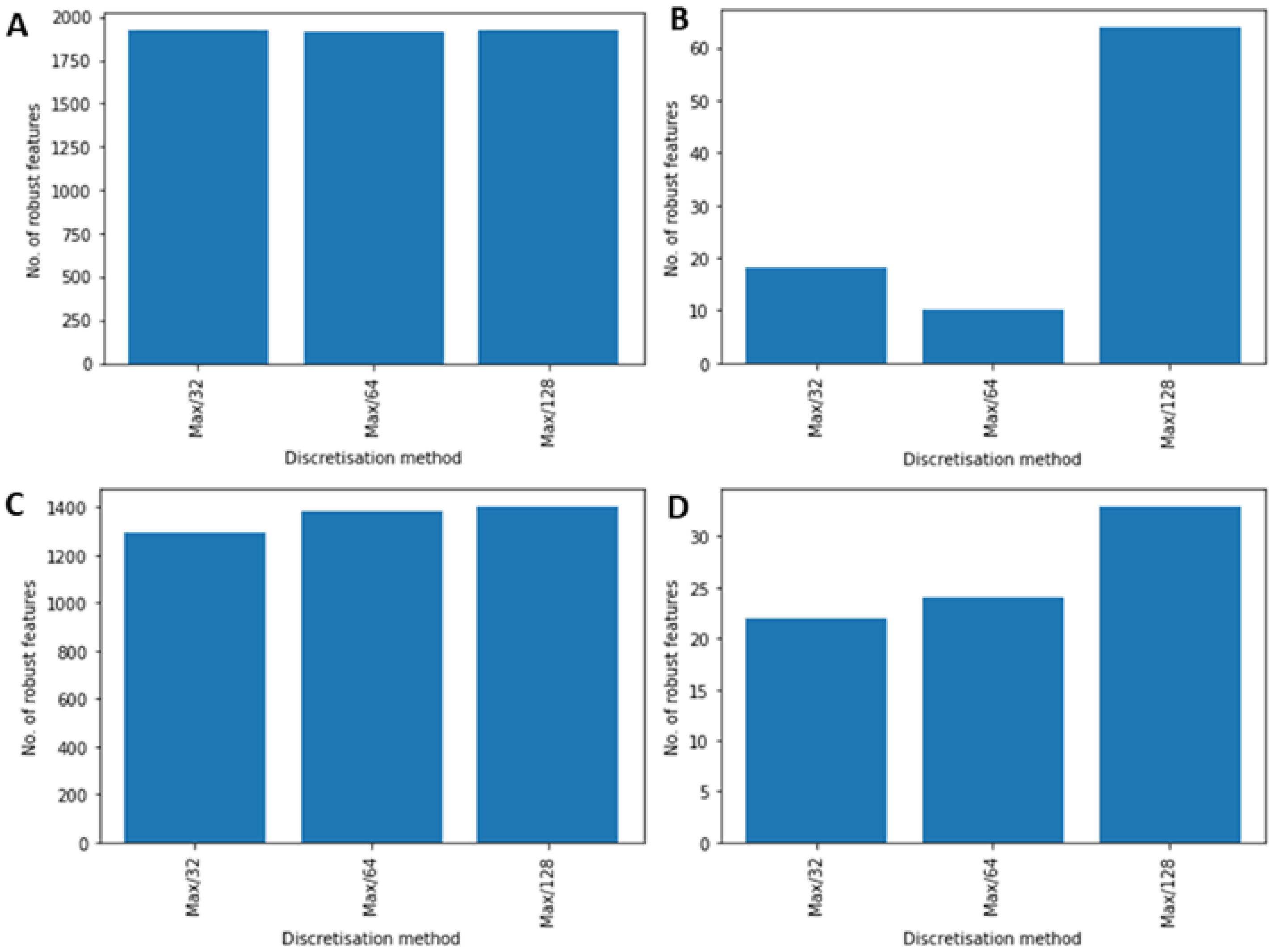

Supplementary Figure 2

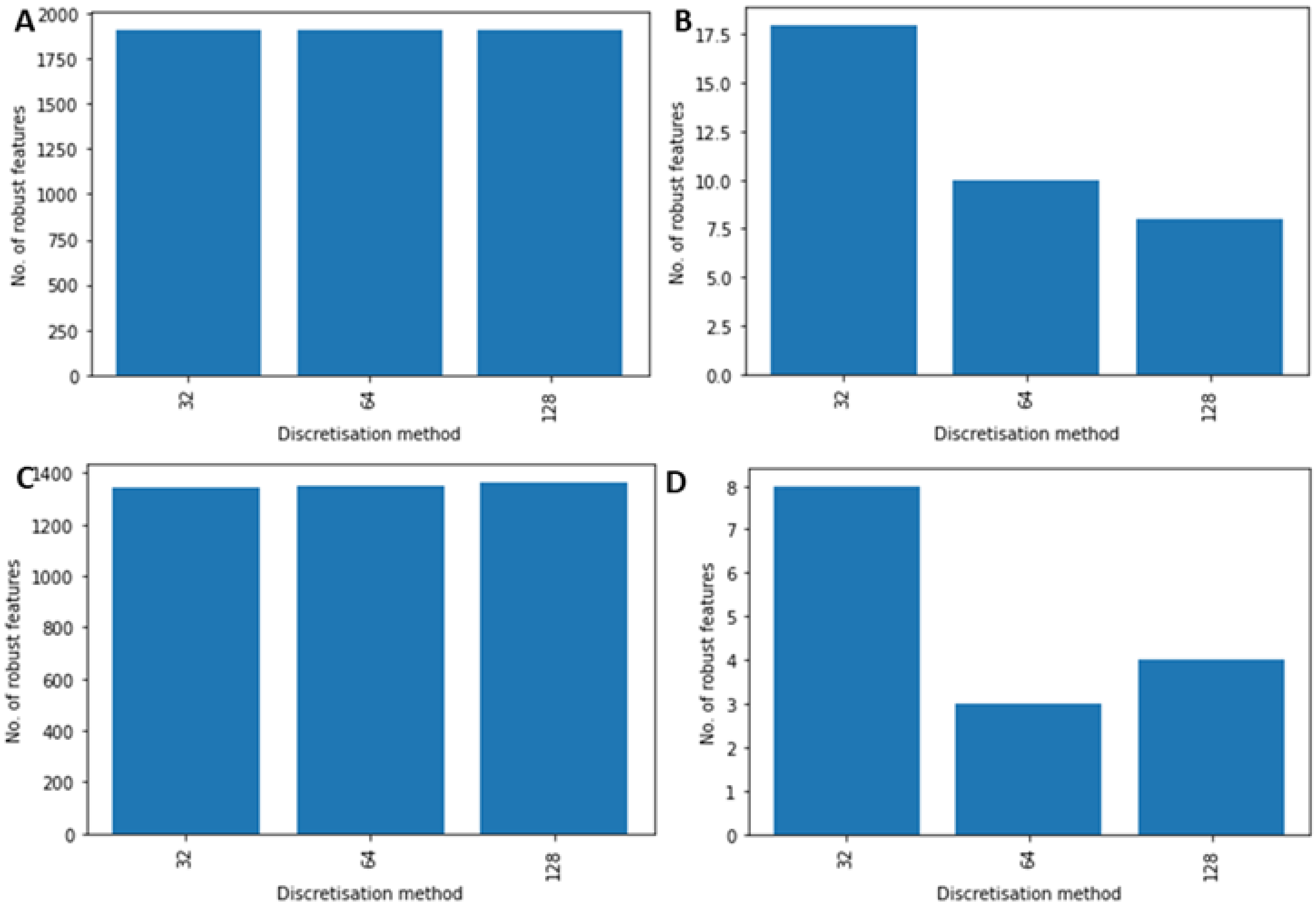

Supplement: Supplementary file 1 — (PDF 540 kb) [file 330_2022_9039_MOESM1_ESM.pdf]
